# Impact of the 1st and 2nd Wave of the COVID-19 Pandemic on Primary or Revision Total Hip and Knee Arthroplasty—A Cross-Sectional Single Center Study

**DOI:** 10.3390/jcm10061260

**Published:** 2021-03-18

**Authors:** Sebastian Simon, Bernhard J.H. Frank, Alexander Aichmair, Philip P. Manolopoulos, Martin Dominkus, Eva S. Schernhammer, Jochen G. Hofstaetter

**Affiliations:** 1 Michael Ogon Laboratory for Orthopaedic Research, Orthopaedic Hospital Vienna-Speising, Speisinger Straße 109, 1130 Vienna, Austria; sebastian.simon@oss.at (S.S.); bernhard.frank@oss.at (B.J.H.F.); alexander.aichmair@oss.at (A.A.); philip.manolopoulos@oss.at (P.P.M.); 2 2nd Department, Orthopaedic Hospital Vienna-Speising, Speisinger Straße 109, 1130 Vienna, Austria; martin.dominkus@oss.at; 3 School of Medicine, Sigmund Freud University Vienna, Freudplatz 1, 1020 Vienna, Austria; 4 Department of Epidemiology, Medical University of Vienna, Kinderspitalgasse 15, 1090 Vienna, Austria; eva.schernhammer@meduniwien.ac.at

**Keywords:** COVID-19, pandemic impact, hip and knee arthroplasty

## Abstract

The aim of this study was to evaluate the number of primary and revision total joint arthroplasties (TJA/rTJA) in 2020 compared to 2019. Specifically, the first and the second waves of the COVID-19 pandemic were evaluated as well as the pre-operative COVID-19 test. A cross-sectional single-center study of our prospectively maintained institutional arthroplasty registry was performed. The first COVID-19 wave and the second COVID-19 wave led to a socioeconomic lockdown in 2020. Performed surgeries, cause of revision, age, gender, and American Society of Anesthesiologists-level were analyzed. Preoperative COVID-19 testing was evaluated and nationwide COVID-19 data were compared to other countries. In 2020, there was a decrease by 16.2% in primary and revision TJAs of the hip and knee compared to 2019. We observed a reduction of 15.8% in primary TJAs and a reduction of 18.6% on rTJAs in 2020 compared to 2019. There is an incline in total hip arthroplasties (THAs) and a decline in total knee arthroplasties (TKAs) comparing 2019 to 2020. During the first wave, there was a reduction in performed primary TJAs of 86%. During the second wave, no changes were observed. This is the first study quantifying the impact of the COVID-19 pandemic on primary and revision TJAs regarding the first and second wave.

## 1. Introduction

The outbreak of the corona-virus-disease-2019 (COVID-19), caused by the Severe Acute Respiratory Syndrome Coronavirus 2 (SARS-CoV-2) in 2019, focused all resources on the coronavirus disease, placing an additional burden on healthcare systems worldwide [1]. The first wave of the COVID-19 pandemic arose in Europe and America during March and April 2020. The second wave, in the fall/winter of 2020, had a substantial higher case number compared to the first one [2,3].

The COVID-19 pandemic has an enormous direct and indirect impact on all healthcare systems [4,5]. Elective surgeries were cancelled, cancer patients endured a delay in care, treatment and screening and even trauma patients had a delay in surgical treatment [6,7,8]. Substantial cancellations and delays of a large number of total hip arthroplasties (THAs) and total knee arthroplasties (TKAs) was observed during the pandemic [9,10]. So far, only general strategies and estimations about the impact of COVID-19 have been published [10,11,12]. However, the COVID-19 pandemic had a substantial impact on orthopedics in 2020. Thus, there is an urgent need for detailed analyses on the COVID-19 pandemic impact on knee and hip arthroplasty in 2020.

Austria gained international attention as a potential center of coronavirus spread due to its geographical location in the middle of Europe and its high incidence rates of COVID-19 during the second wave, with a daily maximum of 106 new cases per 100,000 inhabitants [13]. Due to rising numbers of disease cases, the Austrian government imposed three socioeconomic lockdowns in March, November, and December 2020.

The aim of the present study was to analyze the number of primary and revision total hip and knee arthroplasty performed in 2020 compared to 2019 at the Orthopedic Hospital, Vienna-Speising, Austria, which implants, on average, over 2800 primary and revision TKAs and total joint arthroplasties (TJAs) per year. We evaluated the results of our preoperative COVID tests in 2020.

## 2. Materials and Methods

### 2.1. Study Population

This cross-sectional single center study was approved by the Austrian, Vienna Vinzenz group Ethical Review Board (EK 56/2020). The study period was defined as the time between 1 January 2019 and 31 December 2020. Patients who consecutively underwent primary or revision total joint arthroplasty (TJAs/rTJAs) of the hip or knee joint were included. All surgeries were performed at a single orthopedic tertiary care center in Vienna. Overall, 5268 patients (2866 from 2019 and 2402 from 2020) were included in this study. Patient data were retrieved from a prospectively maintained institutional arthroplasty registry, which comprises information on patient age, gender, American Society of Anaesthesiologists (ASA)-level, and type of surgery.

### 2.2. Institutional COVID-19 Screening

**Figure 1:**
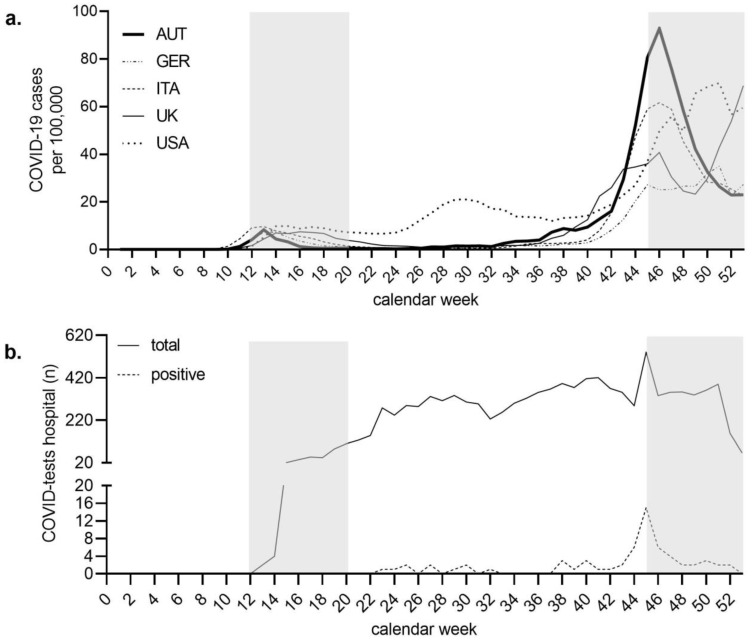
COVID-19 cases and performed tests. (**a**): Daily new cases per 100,000 per calendar week for Austria (AUT), Germany (GER), Italy (ITA), the UK, and the USA. The grey shaded areas visualize the first and the second wave in Austria. (**b**): Absolute number of performed and positive tests in our center per calendar week. The grey shaded areas visualize the first and the second wave in Austria.

According to an institutional standard, on 22 March 2020, all patients undergoing total joint replacement were preoperatively screened for COVID-19 using polymerase-chain-reaction (PCR) tests. Since calendar week 46, screening was performed using a SARS-CoV-2 Rapid Antigen (Ag) Test (Abbott-Panbio™, Fairmed^®^, Zug, Switzerland,). A positive Ag test was confirmed by an additional PCR test. Patients who tested positive for SARS-CoV-2 were not admitted and their operations were postponed (Figure 1b).

### 2.3. Austrian COVID-19 Data

Sources for data retrieval were the European Center for Disease Prevention and Control (ECDC) and the COVID-19 data repository of the Center for Systems Science and Engineering (CSSE) at the Johns Hopkins University. This data is sourced from governments and national and subnational agencies across the world. Daily numbers of SARS-CoV-2 cases were retrieved from https://ourworldindata.org/coronavirus-source-data (last accessed: 25 January 2021). For the purpose of the recent study, the data was presented as daily incidence per 100,000. The mean of daily new cases were calculated for each calendar week. For the maximum evaluation, we took the daily maximum of the contributing calendar week.

The distribution of incident COVID-19 cases by gender was retrieved from Austria’s governmental COVID-19 dashboard for public information, as published and updated daily on https://info.gesundheitsministerium.at (last accessed: 25 January 2021) by the Austrian Federal Ministry of Social Affairs, Health, Care, and Consumer Protection (AGES).

Austria experienced two COVID-19 waves in 2020. In this study, the two waves were each defined as eight-week time periods. The first wave arose between calendar weeks 12–20 and the second wave between calendar weeks 45–53. As a result, the Austrian government imposed three socioeconomic lockdowns. The first one during SARS-CoV-2 wave one, between calendar weeks 12–20, had a duration of eight weeks, and the second and third during SARS-CoV-2 wave two, between calendar weeks 45–49 and 52–54, had a duration of five and three weeks, respectively.

### 2.4. Statistical Analyses

We employed descriptive statistics, using means (M), standard deviations (SD), median (Md), interquartile and minimum (min), and maximum (max) ranges to present continuous study parameters and frequencies and percentages for categorical variables.

We calculated means of the number of performed surgeries (TKAs, THAs, revision total knee arthroplasties (rTKAs), and revision total hip arthroplasties (rTHAs)) in 2019 and 2020 per each calendar week, 1–53. The ratio of revision to primary surgery was also calculated, and causes for revision surgery were summarized for each period and year.

Comparing study groups, Mann–Whitney *U*-testing for metric variables was conducted. For nominally scaled variables, we used chi-squared testing based on crosstabs; *p*-values < 0.05 were considered statistically significant. All analyses were performed using IBM-SPSS^®^ version 25 (Armonk, NY, USA).

## 3. Results

### 3.1. COVID-19 Demographics

Countries such as Austria, Germany, Italy, the United Kingdom (UK), and the United States of America (USA) have been strongly affected by the SARS-CoV2 pandemic. In Austria, the daily new SARS-CoV2 cases peaked with a maximum of 15 per 100,000 inhabitants during the first wave and 100 per 100,000 inhabitants during the second wave. In comparison, in Germany, Italy, the UK, and the USA, the first wave had a peak between 8–11 daily new SARS-CoV2 cases per 100,000 inhabitants and the second wave had a peak between 60–80 daily new SARS-CoV2 cases (Figure 1a).

The maximum peak of daily new cases during the first wave peaked at 14.7 per 100,000 during calendar week 13. The maximum peak of daily new cases during the second wave peaked at 106.4 per 100,000 during calendar week 46 (Figure 1a).

**Figure 2:**
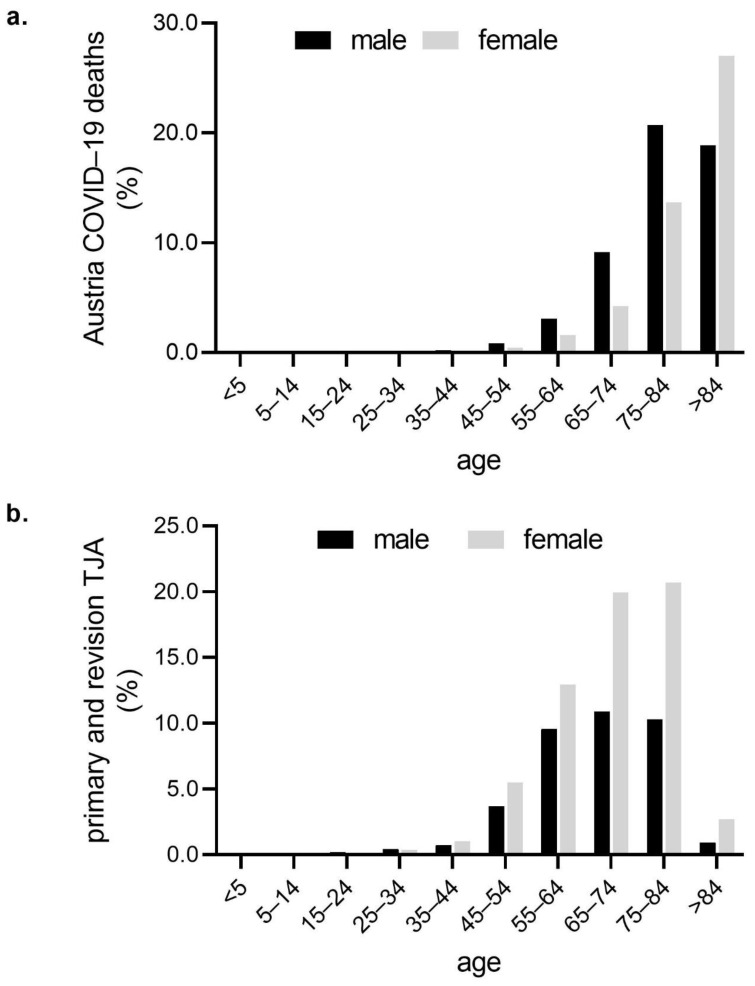
COVID-19 deaths and relative number of performed primary and revision total joint arthroplasties (TJAs). (**a**): Relative number of COVID-19 deaths in Austria per age group. (**b**): Relative number of primary and revision total joint arthroplasties (TJAs) per age group.

In total, there were 361,441 (51.4% female and 48.6% male) SARS-CoV2 cases reported in Austria in 2020. AGES defines COVID-19 deaths as: “COVID-19 death is defined —for surveillance purposes—as one laboratory-confirmed case of COVID-19 resulting in death, where the status recovered has not been present between the status disease and the status death”. There were 6238 (47.1% female and 52.9% male) COVID-19 deaths, which results in a mortality rate of 1.7%. Overall, 16.8% of SARS-CoV2 patients were over 65 years old, but 93.7% of COVID-19 deaths occurred in patients over 65 years old, equaling a mortality rate of 9.5%. In total, 65.5% of our patients were over 65 years. The detailed gender and age distribution of COVID-19 cases and patients is illustrated in Figure 2a,b.

### 3.2. Institutional Results

**Figure 3:**
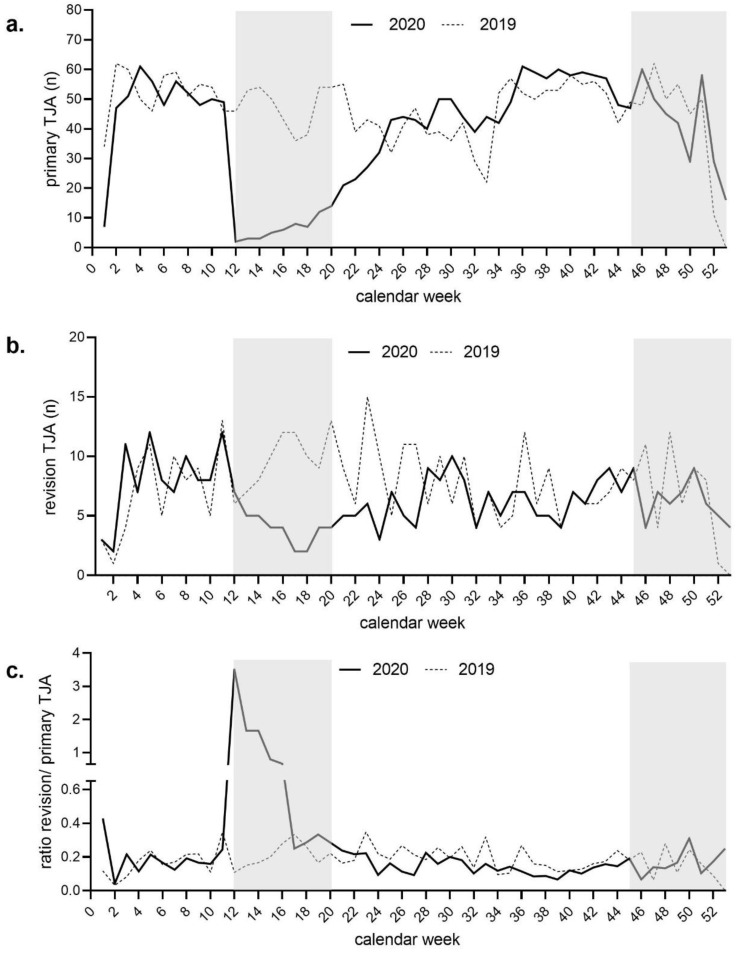
Number of performed primary and revision total joint arthroplasties. (**a**): Absolute numbers of primary TJAs per calendar week, comparing 2019 and 2020. The grey shaded areas visualize the first and the second wave in Austria. (**b**): Absolute numbers of primary TJAs per calendar week, comparing 2019 and 2020. The grey shaded areas visualize the first and the second wave in Austria. (**c**): Ratio between the numbers of revision and primary TJAs per calendar week, comparing 2019 and 2020. The grey shaded areas visualize the first and the second wave in Austria.

In 2020, there was a total reduction of 16.2% (*n* = 464) in primary and revision TJAs of the hip and knee compared to 2019 (2402 vs. 2866). Moreover, a reduction of 15.8% (*n* = 388) in primary TJAs (2457 vs. 2069) and 18.6% (*n* = 76) in revision TJAs (409 vs. 333) was observed between 2019 and 2020, as illustrated in Figure 3a,b. There was a significant relative incline (*p* < 0.010) of THA in 2020 compared to 2019 and a significant relative decline (*p* < 0.049) of TKA in 2020 compared to 2019 (Table 1).

While the number of performed rTJAs remained relatively stable during the study period, the mean ratio of revision vs. primary surgery increased from 0.17 (1.7 revisions vs. 10 primary) in 2019 to 0.31 (3.1 revisions vs. 10 primary) in 2020 (Figure 3c). The causes of rTKAs did not change, while, for rTHAs, the number of septic revisions significantly decreased (*p* < 0.002) and the aseptic loosening revisions significantly increased (*p* < 0.015) during 2020 (Table 1). There were significantly higher ASA-levels in rTJAs (*p* < 0.001), rTKA (*p* = 0.049), and rTHA (*p* = 0.001) in 2019 compared to 2020. Overall, there was no significant difference in age and gender between 2019 and 2020 (Table 1).

During the first COVID-19 wave in 2020, a total of 60 primary TJAs were performed, compared to 428 in the corresponding weeks of 2019, representing a reduction of 86.0% (*n* = 368). The steepest decline in performed primary TJAs during the first COVID-19 wave was noted from calendar week 11 (*n* = 49) to calendar week 12 (*n* = 2), indicating a drop by 96.0% from one week to the other (Figure 3a). The number of rTJAs dropped by 31.0% (*n* = 27). At the same time, the ratio of revision to primary surgery peaked at 3.5 (35 revisions vs. 10 primary). The overall ratio of revision to primary surgery during the first lockdown was 1.15 (11.5 revisions vs. 10 primary) (Figure 3a–c).

During the second COVID-19 wave, a total of 376 primary TJAs were performed, compared to 370 in the corresponding weeks of 2019, resulting in an increase of 1.6% (*n* = 6). The number of rTJAs dropped by 3.4% (*n* = 2). In parallel, the ratio of revision vs. primary surgery peaked at 0.25 (2.5 revisions vs. 10 primary) in calendar week 53. The ratio of revision vs. primary surgery throughout the entire duration of the second wave was 0.17 (1.7 revisions vs. 10 primary) (Figure 3a–c). In our center, a total of 10,635 COVID-19 tests were performed in 2020. Of these, 63 (0.6%) had a positive result (Figure 1b). The overall test data from Austria showed a 9.7% positive test rate in 2020.

## 4. Discussion

This is the first study giving detailed information about the impact of the COVID-19 pandemic on a public tertiary care orthopedic center in 2020, concerning TJAs and rTJAs of the hip and knee joint. We analyzed the number of patients in 2020 compared to 2019. In 2020, a reduction of 16.2% of primary and revision TJAs of the hip and knee occurred, compared to 2019.

The significant differences between the relative incline of THAs and the relative decline of TKAs in 2020 compared to 2019 suggest that a painful hip is more debilitating than a painful knee. Due to the incline of THAs and the decline of TKAs, there is no significant difference in the total number of TJAs. The decline of rTJAs in 2020 is as a result of the reduction of performed primary TJAs in 2020. Between 2019 and 2020, no changes in institutional standard treatment protocol were made. Therefore, the observed changes between 2019 and 2020 in the relative amount of septic and aseptic loosening revisions are not entirely clear and need further research. Moreover, there was a significantly lower ASA-level in rTJAs, rTKAs, and rTHAs in 2020. This could be explained by the fact that only COVID-19 symptom-free and healthy patients underwent elective revision surgery.

Countries worldwide had instituted either full or partial lockdowns. Austria, a country with a population of approximately 9 million, gained international attention for its early coronavirus spread during the first wave and high local incidence rates of COVID-19 during the second wave of the pandemic [14]. The COVID-19 pandemic has affected all aspects of surgical procedures and scheduling, where elective cases were postponed during the first wave of the SARS-CoV-2 pandemic [15,16,17]. Patients suffered from a care delay, and the surgical volume for cancer surgeries and emergency and general surgery procedures, as well as coronary syndrome interventions, dropped substantially during the first wave of the pandemic [7,8,18,19]. In the study by Brown et al., comparing 15 orthopedic institutions in the USA between May and June 2020, 86% of knee and hip arthroplasty operations were postponed or cancelled [20]. All those findings match to the 86% (-368 primary TJAs) decline during the first wave of surgical procedures described in this study. So far, there is no literature available, neither for the detailed numbers of cancellation during the whole SARS-CoV-2 pandemic, nor for the comparison of the first and second SARS-CoV-2 wave in 2020.

There are different reasons for postponed patients waiting for TJAs during the pandemic. During the first wave of the COVID-19 pandemic the federal ministry for Social Affairs, Health, Care, and Consumer Protection of Austria announced their intention to preserve personal protective equipment (PPE) supplies for the care of patients with COVID-19, followed by a massive reduction of elective surgeries. It also enabled recovery areas in operation theatres to be repurposed as overflow intensive care units (ICUs). As our center was part of this plan, post COVID-19 patients were admitted for their recovery. Patient uncertainty, a short preparatory period, and the impact of lockdown messages and advice regarding self-isolation were other reasons for the decline of 368 primary TJAs during the first wave in 2020 compared to the corresponding weeks of 2019. Therefore, the ratio of revision vs. primary surgery increased.

During the second wave, governmental and hospital managements were better prepared regarding the amount of PPE and strategies for the care of COVID-19 and non-COVID-19 patients. During the whole COVID-19 pandemic, our tertiary care center was declared a COVID-19 free institution. When a patient tested positive for SARS-CoV-2, they were not admitted and the scheduled surgery was postponed. Moreover, our center followed the guidelines from the European Society of Sports Traumatology, Knee Surgery, and Arthroscopy (ESSKA) for resumption of orthopedic services. Elective surgery should be performed in COVID-free facilities, hospital stay should be as short as possible, and surgeries should be postponed at the slightest suspicion of a SARS-CoV-2 infection [21,22]. Furthermore, safety guidelines, such as patient and staff weekly testing strategies, as well as the mandatory use of filtering facepiece masks (FFP-2) throughout the facilities were implemented. Most patients undergoing elective surgical procedures do not need ICU resources, and the rapid turnover of elective surgery cases also minimizes the extra pressure on the already strained resources [17]. Therefore, there was almost the same amount of primary TJAs during the second wave in 2020 and the corresponding weeks of 2019.

Regular surgeries recommenced after the cessation of the first lockdown and the relevant recommendations of the Austrian Ministry for Health. It took more than three months after the reduction of TJAs to approach pre-pandemic conditions with regard to TJAs. This is a much shorter time-period than the estimations performed from Jain et al. in US calculate scenarios, in which it would take approximately 7–16 months until the health-care system would be able to revert to a 90% pre-pandemic forecasted volume [23].

The low positive rate (0.6%) of performed SARS-CoV-2 tests in our center is another reason to pursue an elective surgery program. One reason for the low positive rate in our center (0.6%) compared to the overall SARS-CoV-2 test positive rate of Austria (9.7%) is that only COVID-19 symptom-free and healthy patients underwent testing following elective surgery.

Translational research results from the previous wave/s are required to prepare for a potential third wave, but also for other pandemics in the future. The main limitation of this study is its single-center design. The COVID-19 impact on a tertiary care institution will not represent the impact on other orthopedic divisions. Multi-center studies are needed to evaluate the total impact of the COVID-19 pandemic on health care systems and assess the amount of the total backlog and the possible changes in patient’s mortality and morbidity.

## 5. Conclusions

Our study demonstrated that the COVID-19 pandemic has had a substantial impact on a public orthopedic tertiary care center, especially during the first COVID-19 wave. Adopting specific strategies for continuing elective surgery, ensuring, at the same time, patient and personnel safety, may allow care centers to perform TJAs at the same pre-pandemic conditions.

## Figures and Tables

**Table 1:** Characteristics of study relevant parameters for surgical procedures in 2019 and 2020 including gender, age, and American Society of Anaesthesiologists (ASA)-level. Frequencies and column percentage of distribution according occurrence of septic, aseptic loosening, Instability/Dislocation, fracture, other septic conditions, abrasion, pain, implant failure, and leg length difference (LLD); ** *p* ≤ 0.01, * *p* ≤ 0.05.

Parameter	2019	2020	*p*-Value
**Total TJAs**	***n* = 2866**	***n* = 2402**	
Primary TJAs total	2457 (85.7%)	2069 (86.1%)	0.672
Gender female	1552 (63.2%)	1330 (64.3%)	0.309
male	905 (36.8%)	739 (35.7%)	
Age	68.1 (±11.4)	68.2 (±11.3)	0.360
ASA-level	1.97 (±0.46)	1.96 (±0.47)	0.420
Revision TJAs total	409 (14.3%)	333 (13.9%)	0.672
Gender female	247 (60.4%)	191 (57.4%)	0.923
male	162 (39.6%)	142 (42.6%)	
Age	69.3 (±11.7)	68.5 (±12.7)	0.485
ASA-level	2.21 (±0.53)	2.02 (±0.46)	<0.001 **
**Total TKAs**	***n* = 1477**	***n* = 1147**	
Primary TKAs	1272 (86.1%)	956 (83.3%)	0.049 *
Gender female	827 (65.0%)	620 (64.9%)	0.937
male	445 (35.0%)	336 (35.1%)	
Age (years)	69.6 (±9.5)	69.6 (±9.7)	0.751
ASA-level	2.02 (±0.42)	2.01 (±0.41)	0.642
Revision TKAs	205 (13.9%)	191 (16.7%)	0.049 *
Gender female	123 (60.0%)	114 (59.7%)	0.915
male	82 (40.0%)	77 (40.3%)	
Age (years)	71.2 (±9.5)	69.9 (±10.7)	0.320
ASA-level	2.18 (±0.44)	2.07 (±0.41)	0.048 *
Cause of Revision TKAs			
Septic	95 (46.3%)	88 (46.1%)	0.519
Instability	35 (17.1%)	33 (17.3%)	0.957
Pain	16 (7.8%)	24 (13.0%)	0.748
Aseptic loosening	28 (13.7%)	21 (11.0%)	0.276
Fracture	6 (2.9%)	8 (4.2%)	0.252
Other aseptic	14 (6.8%)	8 (4.2%)	0.687
Wear	7 (3.4%)	5 (2.6%)	0.852
Implant Failure	4 (2.0%)	4 (2.1%)	0.919
**Total THA**	***n* = 1389**	***n* = 1255**	
Primary THAs	1185 (85.3%)	1113 (88.7%)	0.010 **
Gender female	725 (61.2%)	710 (63.8%)	0.197
male	460 (38.8%)	403 (36.2%)	
Age (years)	66.4 (±12.9)	66.8 (±12.4)	0.582
ASA-level	1.92 (±0.50)	1.91 (±0.51)	0.715
Revision THAs	204 (14.7%)	142 (11.3%)	0.010 **
Gender female	124 (60.8%)	90 (63.4%)	0.625
male	80 (39.2%)	52 (36.6%)	
Age (years)	67.4 (±13.3)	66.6 (±14.9)	0.320
ASA-level	2.23 (±0.60)	1.97 (±0.51)	0.001 **
Cause of Revision THAs			
Septic	95 (46.6%)	43 (30.3%)	0.002 **
Aseptic loosening	50 (24.5%)	52 (36.6%)	0.015 *
Dislocation	26 (12.7%)	24 (16.9%)	0.279
Fracture	14 (6.8%)	16 (11.3%)	0.152
Other aseptic	4 (2.9%)	2 (1.4%)	0.699
Wear	2 (1.0%)	2 (1.4%)	0.714
Pain	2 (1.0%)	1 (0.7%)	0.146
Implant Failure	4 (2.0%)	1 (0.7%)	0.221
LLD	2 (1.0%)	1 (0.7%)	0.785

TJA, total joint arthroplasty; THA, total hip arthroplasty; TKA, total knee arthroplasty.

## Data Availability

Daily numbers of SARS-CoV-2 cases were retrieved from https://ourworldindata.org/coronavirus-source-data (last accessed: 25 January 2021). The distribution of incident COVID-19 cases by gender was retrieved from Austria’s governmental COVID-19 dashboard for public information, as published and daily updated on https://info.gesundheitsministerium.at by the Austrian Federal Ministry of Social Affairs, Health, Care, and Consumer Protection (last accessed: 25 January 2021).
